# Comparison of Return-to-Sports Rates Between Male and Female Australian Athletes After ACL Reconstruction

**DOI:** 10.1177/23259671231169199

**Published:** 2023-06-02

**Authors:** Haydn J. Klemm, Julian A. Feller, Kate E. Webster

**Affiliations:** * OrthoSport Victoria, Epworth HealthCare, Melbourne, Australia; † School of Allied Health, Human Services and Sport, La Trobe University, Melbourne, Australia; Investigation performed at OrthoSport Victoria and La Trobe University, Melbourne, Australia

**Keywords:** anterior cruciate ligament reconstruction, female athletes, knee, male athletes, patient outcomes, return to sports

## Abstract

**Background:**

Return to sports (RTS) is a goal for most patients who undergo anterior cruciate ligament reconstruction (ACLR). Although it has been reported that women RTS at a significantly lower rate compared with men, demographic and contextual factors that may be associated with this have not been investigated.

**Purpose:**

To compare RTS rates between men and women and investigate factors that may be associated with different rates of RTS in an Australian context.

**Study Design:**

Cohort study; Level of evidence, 3.

**Methods:**

A total of 1338 patients who underwent primary ACLR between January 2014 and December 2017 were invited to complete a detailed sports participation questionnaire 2 to 7 years after surgery. RTS rates were calculated and compared between men and women overall and after stratifying by age at surgery (<20, 20-29, 30-39, or ≥40 years) and geographical location (metropolitan or rural). Contingency analysis was performed to compare factors associated with rates of RTS.

**Results:**

The survey completion rate was 81% (1080/1338). Overall, women had a significantly lower RTS rate compared with men (65.4% vs 74.9%; *P* = .001). However, when patients were grouped by age, the lower rate of RTS for women was significant only in the 20- to 29-year age group (*P* = .01). For athletes who returned to sports, there was no sex-based difference when comparing the levels of RTS. When grouping patients based on geographical location, there was a significantly lower rate of RTS in metropolitan-based women compared with metropolitan-based men (*P* < .001) and rural-based women (*P* = .042).

**Conclusion:**

Although women returned to sports at a lower rate than men overall, this difference was predominantly seen in the 20- to 29-year age bracket and in those who lived in metropolitan areas. There was no difference between men and women regarding the RTS level.

Most patients who undergo anterior cruciate ligament (ACL) reconstruction (ACLR) do return to sports (RTS). There are, however, a myriad of factors that can affect whether a patient returns to sports after an ACLR, some of which are as follows: physical factors, such as the condition of the knee; psychological factors, most notably fear of reinjury; the specific sport to which the patient wishes to return; social factors, such as work and family commitments; and subsequent injury to either knee.^3,15^ Within the ACLR patient population, women have reported RTS at lower levels compared with men.^1,14,18^ This lower rate of return has been found using different methods of assessing RTS. In a meta-analysis, Ardern et al^1^ found that women returned to sports at a significantly lower rate than men both in terms of a return to preinjury level of sport (52% vs 61%; *P*≤ .01) and a return to a competitive level of sport (68% vs 78%, *P*≤ .01). Another meta-analysis^14^ found that women had a lower Tegner score and a 12% increased pooled relative risk of failing to RTS. An interaction between age and sex has also been found, with lower RTS rates reported in women compared with men who are ≤35 years but no sex difference in RTS rates after the age of 35 years.^18^ No demographic factors other than age have been reported that may explain why women may RTS at a lower rate than men. While this lower rate of RTS in women has been shown to exist, it has not yet been fully described outside of broad age groups. A focused analysis would allow for a more thorough understanding of this finding.

This study aimed to compare RTS rates by sex in Australian athletes and identify factors that may contribute to any differences between men and women in RTS rates after ACLR. It was hypothesized that the RTS rate would be lower in women than men and vary according to age.

## Methods

### Patients

The protocol for this study received ethics committee approval. Eligible patients were identified from a practice database of 4 experienced knee surgeons at a private metropolitan clinic in Melbourne, Australia. This database consisted of patients who had undergone their first primary ACLR surgery between January 2014 and December 2017.

The study inclusion criteria were having had a primary ACLR surgery using an autograft, having participated in sports before their injury, and having indicated an intention to RTS after surgery. The exclusion criteria were as follows: a previous contralateral ACLR; bilateral ACLR; ACLR involving reconstruction or repair of another ligament, or any previous open knee surgery; and not participating in sports at the time of ACL injury.

### Surgical Details

All ACLR procedures were arthroscopically assisted. Three different grafts were used, including hamstring, patellar, and quadriceps tendon, with the default graft being a hamstring tendon. The decision to use a graft other than the hamstring tendon was made by the patient and treating surgeon after a discussion of the relative merits and disadvantages of each graft type. For all graft types, femoral fixation using an Endobutton (Smith & Nephew) and tibial fixation using a metal interference screw were performed, regardless of graft type.

All patients underwent the same rehabilitation protocol, with a focus on early recovery of full active knee extension and quadriceps function. Weightbearing was allowed on an as-tolerated basis from the first postoperative day. No braces or splints were used. Progression was guided by the presence and degree of pain and swelling. All patients were provided with the same rehabilitation protocol as detailed by Batty et al.^2^ The minimum requirements for RTS clearance from the treating surgeon were as follows: no effusion; an essentially full range of motion; good quadriceps strength with good control of a single-leg squat; and at least 4 weeks of unrestricted training. These minimum requirements were not assessed as part of a formalized RTS test battery.

### Data Collection

Participants were asked to complete a preoperative questionnaire that asked them to indicate their main preinjury sports, self-select their level (professional athlete, high-level competitive sports, frequent sports, sports sometimes, and nonsporting), specify the frequency of sport participation before their ACL injury, and indicate whether they intend to RTS after their surgery.

Participants were sent an electronic survey that included questions related to RTS between 2 and 7 years after surgery. These questions asked patients to report whether they had returned to sport and at any level (no return, return to training, return to play at a lower level, or return to the same or higher level). Participants who stated they had not returned to sport were asked whether they still plan to RTS, and if they reported not planning to RTS, they were given categories to choose from (“No, because I’m worried about injuring my knee again,”“No, because my knee doesn’t feel up to it,” or “No, because of reasons other than my knee.”). At least 2 further phone calls were made to those participants who did not complete the survey on the first request. Medical records of the cohort were reviewed to confirm details, including demographic characteristics and surgical information.

### Data and Statistical Analysis

For analysis, participants were categorized according to sex, age at the time of surgery (<20, 20-29, 30-39, or ≥40 years), and geographical location based on the postcode of their residence (metropolitan or rural). The location was considered metropolitan if the postcode was within the boundaries of metropolitan Melbourne and rural if the postcode was outside of those boundaries. RTS data were categorized in a binary fashion (yes or no) as well as by the RTS level (lower, same, or higher level). Patients who stated that they had only returned to training were categorized as not having returned to sports.

Groups were compared with the chi-square test using SPSS Version 28 (IBM) software. *P* < .05 was used to indicate statistical significance.

## Results

Of the eligible 1338 patients, there were 1080 participants for whom RTS data were collected at least 2 years postoperatively, with a follow-up rate of 81%. Participants included 658 men (60.9%) and 422 women (39.1%). The mean follow-up time was 3 ± 1.02 years after surgery. Table 1 shows the level of sports played by the study participants, and Table 2 summarizes the surgical details.

**Table 1:** Sporting Level Before Injury (N = 1080 Patients)

	No. of Participants (%)
Professional athlete	41 (3.8)
High-level competitive sports	363 (33.7)
Frequent sports	584 (54.1)
Sports sometimes	91 (8.4)

**Table 2:** Surgical Details (N = 1080 Patients)

	No. of Participants (%)
Graft type	
Hamstring tendon	995 (92.1)
Patellar tendon	25 (2.3)
Quadriceps tendon	59 (5.5)
Contralateral patellar tendon	1 (0.1)
Additional lateral extra-articular tenodesis	11 (1)
Medial meniscal injury and treatment	
No tear	770 (71.3)
Stable tear: no treatment	44 (4.1)
Tear: partial resection	148 (13.7)
Tear: repair	100 (9.3)
Previous partial resection	18 (1.7)
Lateral meniscal injury and treatment	
No tear	698 (64.7)
Stable tear: no treatment	131 (12.1)
Tear: partial resection	201 (18.6)
Tear: repair	42 (3.9)
Tear: repair and partial resection	5 (0.5)
Previous partial resection	3 (0.3)
Chondral injuries	
Patellofemoral compartment	147 (13.6)
Medial compartment	143 (13.2)
Lateral compartment	79 (7.3)

A total of 769 participants (71.2%) reported RTS between 2 and 7 years after ACLR. A significantly higher proportion of men (n = 493 [74.9%]) returned to sports compared with women (n = 276 [65.4%]) (*P* = .001). After stratifying by age group, women had a lower RTS rate than men in all age groups; however, a significant difference was found only in the 20- to 29-year age group (*P* = .01) (Table 3). Participants in the 20- to 29-year age group were split into those aged 20 to 24 years and those aged 25 to 29 years, and a comparison was made between women and men. In both groups, women had a significantly lower RTS rate compared with men (20-24 years, 62% v 75% [*P* = .025]; 25-29 years, 62% v 79%; [*P* = .011]).

**Table 3:** Patients Who Returned to Sports by Age Group and Sex (n = 769)^*a*^

Age Group, y	Women	Men
<20	110/140 (79)	136/162 (84)
20-29^*b*^	90/145 (63)	243/315 (77)
30-39	47/78 (61)	71/113 (64)
≥40	29/59 (49)	43/68 (65)

a Data are reported as No. of participants who returned to sports/overall group sample size (%).

b Significant differences between men and women (*P* = .01).

Of the 769 participants who had returned to sport, the RTS level was available for 621 patients—374 men (60.22%) and 247 women (39.77%). There was no significant difference between men and women for the RTS level at any age group (Table 4).

**Table 4:** RTS Levels in Patients by Age Group and Sex (n = 621)^*a*^

RTS Level	Women	Men
Age <20 y		
Same/higher	76 (82)	90 (82)
Lower	17 (18)	20 (18)
Age 20-29 y		
Same/higher	57 (69)	137 (70)
Lower	26 (31)	30 (30)
Age 30-39 y		
Same/higher	27 (63)	43 (75)
Lower	16 (37)	14 (25)
Age ≥40 y		
Same/higher	19 (68)	28 (70)
Lower	9 (32)	12 (30)

a Data are reported as No. of participants (%). RTS, return to sports.

When participants were categorized based on geographical location, 74% of men and 65% of women lived in a metropolitan postcode (Table 5). Metropolitan-based women returned to sports at a significantly lower rate compared with metropolitan-based men (*P* < .001) and rural-based women (*P* = .042).

**Table 5:** Patients Who Returned to Sports by Geographical Location and Sex (n = 769)^*a*^

Setting	Women^*b*^	Men
Metropolitan^*c*^	170/273 (62)	360/486 (74)
Rural	106/149 (71)	133/172 (77)

a Data are reported as No. of participants who returned to sports/overall group sample size (%).

b Significant differences between women in metropolitan vs rural settings (*P* = .042).

c Significant differences between metropolitan-based women vs men (*P* < .001).

Of the 311 patients who had not returned to sports, data regarding future RTS plans were available for 212 participants (response rate, 68%)—111 men (52.36%) and 101 women (47.64%) (Table 6). There was no significant difference between men and women in their responses across the whole group or in any of the age groups.

**Table 6:** Patients Who Did Not Return to Sports and Their Reasons for Not Returning (n = 212)^*a*^

	Not Planning to Return			
	Worried About Injuring Knee Again	Knee Doesn’t Feel Up to It	Reasons Other Than Knee	Planning to Return
Total				
Women	40/101 (39.60)	14/101 (13.86)	25/101 (24.75)	22/101 (21.78)
Men	39/111 (35.14)	12/111 (10.81)	31/111 (27.93)	29/111 (26.13)
Age <20 y				
Women	4/16 (25)	1/16 (6.25)	5/16 (31.25)	6/16 (37.50)
Men	6/14 (42.86)	1/14 (7.14)	3/14 (21.43)	4/14 (28.57)
Age 20-29 y				
Women	17/43 (39.53)	7/43 (16.28)	9/43 (20.93)	10/43 (23.26)
Men	18/50 (36)	7/50 (14)	15/50 (30)	10/50 (20)
Age 30-39 y				
Women	10/22 (45.45)	3/22 (13.64)	4/22 (18.18)	5/22 (22.73)
Men	9/31 (29.03)	3/31 (9.68)	9/31 (29.03)	10/31 (32.26)
Age ≥40 y				
Women	9/20 (45)	3/20 (15)	7/20 (35)	1/20 (5)
Men	6/16 (37.50)	1/16 (6.25)	4/16 (25)	5/16 (31.25)

a Data are reported as No. of responses/group sample size (%).

## Discussion

This study confirmed that women in Australia returned to sports at a lower rate than men after ACLR. This lower RTS rate was found to be most significant in women in their 20s. No difference between men and women was found in terms of RTS levels. A lower RTS rate was also found among women who were living in a metropolitan location compared with both metropolitan-based men and rural-based women.

The lower RTS rates in women compared with men has been previously reported,^1,14,18^ and the rates in this study are similar. The present study found that overall, women returned to sports at a 9.5% lower rate than men. Previous studies from the same institution reported a 9%, 10%, and 13.2% difference when comparing between sexes return to either the preinjury level, a competitive level, and return to a level 1 sport respectively.^1,18^ However, these previous studies reported RTS rates at a much earlier time point than the present study. The current study importantly confirms that this difference in RTS rates between the sexes remains at a later time point when all patients should have had ample time to return. However, when the factor of age is also considered, it becomes clear that the majority of the difference in RTS rates between men and women is in patients in their 20s. Previous work has shown that RTS rates may differ with different age groupings,^18^ which raises the question of how this may compare with changes in sports participation in the general population.

A recent study,^5^ which reported participation rates using registration data from sports clubs in the same location (Victoria, Australia) and during a similar time frame (2015 and 2019), provided data for comparison. Sports participation data from the study demonstrated not only that women participate in sports at a lower rate compared with men in general, but also that women have a greater decrease in participation after adolescence, specifically between the ages of 20 to 29 years. There is a myriad of factors that may be driving this. Although research has mostly focused on women in adolescence rather than in younger adulthood, it is likely still relevant for comparison. Focus group data from adolescent girls have suggested the following factors for decreased participation in organized sports: lack of flexible schedules; focus on education; lack of confidence in skills; and unsupportive coaches.^7,8,12,13^ In this context, injury has also been noted; however, there is a lack of details in the definition of this as a reason for ceasing sport.^13^ Family-related obligations, such as childbearing and childrearing, may play a part in this shift; however, this was not apparent from the results of this study. Regardless of the cause, the sex differences in RTS rates currently seen in an ACLR population may be mirroring the natural attrition and sex differences that occur in the general population. It is not yet understood whether an ACL injury causes a premature transition away from organized sports, and this warrants further investigation.

When reviewing the responses of participants who had not returned to sports in terms of whether they still planned to RTS, there was no difference between men and women in the whole cohort or any of the age-based groupings. In the whole cohort, the majority of participants reported not returning to sports because of concerns of reinjury, which is consistent with previous studies.^17^ It is worth noting that approximately 20% of participants still reported planning to RTS even at ≥2 years after surgery. However, the response rate for this question was 68%, and therefore our findings may not fully represent the reasons for not returning to sports.

When comparing data between men and women who did RTS, no difference was seen in their RTS levels. Differences between men and women have been reported by Ardern et al^1^ in those returning to the preinjury (9% RTS rate difference) and competitive levels (10% difference). The present study used different categories compared with those previously reported, with participants reporting whether they returned to sports at the same or higher levels or lower levels compared with before their injury, and also categorized participants into age brackets for the analysis. This may explain the difference in the results. The current results indicate that whatever factors cause a lower rate of RTS for women, these factors do not affect the sports level to which they may return.

Geographical location was also a factor that influenced RTS. The lower rate of RTS appeared to be more focused on women who reside in metropolitan areas compared with women who reside in rural areas. This is demonstrated by rural-residing women having an RTS rate that is only 7% lower than men, which is less than the difference between women and men in the metropolitan area (11%; 63% vs 74%) as well as overall (9.5%; 65.4% vs 74.9%).

There is evidence that it is common for sports participation to be higher in regional/rural areas compared with metropolitan areas.^5,9^ In the Australian context, this is attributed to factors of population density and the availability of sports and sporting infrastructure that results in rural communities to be limited to traditional sports compared with a wider range of sports and leisure activities available in metropolitan areas.^6^ This wider range of activities in metropolitan areas results in reduced participation in organized sports, while the lesser range of options in rural areas has been reported to increase participation in organized sports for social benefits.^4,6,7^ While other studies have linked a patient’s geography through their postcode to patient outcomes^10,16^ and comparisons of the epidemiology of ACL injury and treatment have been made using registry data,^11^ there has not been an emphasis on RTS and sex comparisons. An association between a patient’s neighborhood socioeconomic status and their patient-reported outcomes was reported by Jones et al,^10^ with those living in neighborhoods with a lower socioeconomic status having lower scores on the Knee injury and Osteoarthritis Outcome Score, the International Knee Documentation Committee Subjective Knee Evaluation Form, and the Marx activity rating scale.

### Limitations

This study has several limitations. The RTS data were self-reported and thus may not fully capture patients’ RTS as opposed to data from organized competitive sports. While some patients may not RTS, they may participate in activities that involve a strenuous activity that do not take the form of organized competitive or recreational sports. The questions used in the present study would not likely allow for this distinction. Participants who did not RTS and indicated reasons other than their knee were not asked to detail what these “other” reasons were, which did not allow for a comparison between men and women. The type of sport that this population participated in was gendered; for example, the majority of male participants participated in Australian rules football while the majority of female participants participated in netball, and this did not allow for direct comparisons between men and women based on the sport played. This follow-up question should be included in further research. As this study was conducted retrospectively, data relating to social determinants of health, such as household income and education level, were limited; however, this should be included in future research. This study is also specific to the setting it was conducted in—a private metropolitan clinic in Australia—and therefore these findings may not be generalizable to other settings.

## Conclusion

Although women returned to sports at a lower rate than men overall, this difference was predominantly seen in the 20- to 29-year age bracket. For patients who did RTS, there was no sex difference related to their RTS levels. Metropolitan-based women had a lower rate of RTS compared with rural-based women and metropolitan-based men.
